# Global Interdependence, Just Vaccine Allocation, and Compensatory Justice: A New Model

**DOI:** 10.1111/dewb.12486

**Published:** 2025-06-08

**Authors:** Kalen J. Fredette

**Affiliations:** ^1^ Stritch School of Medicine, Loyola University of Chicago Chicago Illinois USA

**Keywords:** compensatory, interdependence, justice, retributive, vaccine allocation

## Abstract

During the COVID‐19 pandemic, numerous models were offered for how scarce vaccine resources should be distributed. Proposed vaccine distribution models generally were divided between nationalist models, which give preference to nationals, and cosmopolitan models, which ignore national boundaries. More defensible international vaccine distribution program proposals incorporate ethical considerations from both cosmopolitanism and nationalist models. To date, however, proposed models have insufficiently considered how global interdependence has resulted in economic and ecological harms by high‐income countries (HICs) against low‐to‐middle income countries (LMICs). Because these harms create health burdens for the populations of LMICs, compensatory justice should impact distribution determinations. This paper argues that adequately factoring in global interdependence, compensatory justice, as well as the disproportionate impact of pandemics on LMICs, just vaccine distribution may require prioritizing LMIC populations over those of HICs.

## 1 Introduction

The COVID‐19 pandemic ushered in a multidisciplinary debate regarding appropriate international vaccine distribution. Often this debate placed discussants into one of two camps: vaccine nationalism and vaccine cosmopolitanism. This paper assesses vaccine nationalism, vaccine cosmopolitanism, and hybrid approaches as candidates for just vaccine distribution models. Hybrid approaches proposed to date have the advantage of accommodating key moral commitments of both cosmopolitan and nationalism, but have neglected to account for moral obligations created by an increasingly interdependent global community. A hybrid vaccine distribution model, which is sensitive to moral obligations created by global interdependence, best captures a nation‐state's distributive justice obligations. This model can expose failures of the COVID‐19 pandemic response and provide guidance for future pandemic vaccine distribution.

## 2 Cosmopolitanism and Vaccine Distribution

Cosmopolitan theorists argue that robust distributive justice obligations transcend national boundaries. According to such theorists, social cooperation and interdependence among individual persons and people groups is the basis for distributive justice claims [1]. Because social cooperation and interdependence do not terminate at national borders, neither do social/moral obligations [1, 2]. Under this view, national boundaries have no moral relevancy in the distribution of resources because nationality is a morally arbitrary consequence of the birth lottery, like gender, race, and ethnicity [3, 4]. Cosmopolitans reason that the equality of persons implicates an equal distribution of resources among persons globally [2, 3, 4]. Inequitable distribution of resources requires specific, morally relevant reasons to justify any departure from equality.

In his provocative and influential essay, “Famine, Affluence and Morality,” the utilitarian Peter Singer helps illustrate several core components of the cosmopolitan view with a simple scenario: Singer considers an individual (who I will refer to as Peter) walking next to a shallow pond who observes a child drowning in it [5]. Presuming that Peter can safely rescue the child, incurring only the “morally insignificant” burdens (e.g., inconvenience, loss of time, and muddy clothes), Peter has a moral obligation to rescue the child. Singer then argues that a similar moral obligation requires relatively affluent persons to extend “rescue” aid to imperiled persons in impoverished nations. Tracking the cosmopolitan position, Singer contends that geography and nationality are morally irrelevant considerations. Singer states that the principle driving the moral obligation to rescue takes:

no account of proximity or distance. It makes no moral difference whether the person I can help is a neighbor's child ten yards from me or a Bengali whose name I shall never know, ten thousand miles away…The fact that a person is physically near to us, so that we have personal contact with him, may make it more likely that we *shall* assist him, but this does not show that we *ought* to help him rather than another who happens to be further away. If we accept any principle of impartiality, universalizability, equality, or whatever, we cannot discriminate against someone merely because he is far away from us (or we are far away from him). (emphasis original) [5, p. 232]

According to Singer, the logistical challenges of delivering aid across geographic distances, which may have once impacted moral obligations to assist, were (as of the article's 1972 publication) obliterated by “instant communication and swift transportation.” Such advances transformed the world into a “global village” and radically changed our “moral situation [5].”

Vaccine cosmopolitans argue that a just distribution of vaccines in response to pandemics should generally result in equitable distribution of vaccines across nation‐states [6]. If epidemiological guidance indicates that frontline medical workers and medically vulnerable populations should be prioritized in a domestic distribution of vaccines, this model should be extended internationally, with frontline medical workers and medically vulnerable populations receiving the vaccine in every country, before less vulnerable persons in any one country [6]. This approach limits transmission among healthcare workers and their patients and, arguably, minimizes the overall global death toll. In response to the COVID‐19 pandemic, one prominent distribution proposal that tracked this approach was the “Fair Priority Model.” (FPM) [7] Advocates of FPM concede that nations would likely prioritize domestic vaccine distribution initially.^1^ However, after initial vaccine distribution, they propose FPM as an “ethical framework” for equitable international vaccine distribution, irrespective of national boundaries, according to three ethical criteria: harm limitation, benefit to the disadvantaged, and recognition of equal concern of persons [7, p. 1312].

## 3 Nationalism and Vaccine Distribution

Proponents of nationalism frequently argue that distributive justice adheres only domestically, that is, within the nation‐state. They argue that the requisite level of social cooperation needed to ground justice claims occurs within domestic communities and does not extend beyond national borders. Nationalists reject a cosmopolitan conception of international distributive justice for one or more of the following reasons: (1) international social cooperation is insufficiently robust and therefore ethically irrelevant compared to cooperation shared by fellow citizens; (2) a domestic community shapes individuals and their moral obligations; (3) and/or necessary institutional redistributive enforcement mechanisms simply do not exist in the international polity.

As to the insufficiency of international social cooperation, an early critic of cosmopolitanism, Brian Barry, states:

Trade, however multilateral, does not constitute a cooperative scheme of the relevant kind. Trade, if freely undertaken, is (presumably) beneficial to the exchanging parties, but it is not, it seems to me, the kind of relationship giving rise to duties of fair play [8].

The arms‐length economic international cooperation presented by Barry can be distinguished from the enmeshed cooperation of fellow citizens, who are collectively bound together socially, economically, legally and politically.

Some nationalists argue that domestic communities create associative ties and obligations that affect moral obligations for individual community members and their leaders. The social interaction/cooperation within various domestic communities creates obligations of reciprocity, reparation, compensation, solidarity, and patriotism.^2^

According to this view, Singer's illustration improperly discounts the moral impact that various types of relationships may have on Peter's moral obligation to give aid. If the drowning child is Peter's family member, related to a friend, a neighbor, or another salient community member, the moral calculation may be impacted. For example, it may impact what level of sacrifice or risk might be required of Peter in a rescue attempt. According to nationalists, while the transactional challenges of geography and communication may have been overcome by technological advances, the description of a “global village” is inaccurate to the extent that it ignores prominent features in the moral landscape of most individuals. Under the nationalist view, it *does* matter if the drowning child is Bengali or from a community that has more moral salience for Peter. Moreover, the nationalist is likely to observe that often, in a resource limited environment, it is not simply a question of whether there is a moral requirement whether to rescue. Rather, an additional question is raised ‐‐ who among multiple potential victims has the strongest moral claim on the potential rescuer. Nationalists who adopt this line of reasoning need not reject the concept of international distributive justice. Rather nationalists conclude that the moral obligations created within a community justify preferential treatment of community members in international distribution of resources [7, p. 1309].

Other proponents of nationalism reason that redistributive justice requires a coercive body like a nation‐state government to enforce justice claims and oversee redistribution, which has no corollary in the international arena [10]. In the absence of a legal structure and an enforcement body, no justice claims can be raised which would permit redistribution to resolve inequality [10, p. 116]. This paper will assume both that no international institution can legally enforce distributive justice claims and yet one can appropriately make a moral claim of distributive injustice even in the absence of a legally enforceable correlative claim.

Vaccine nationalists argue that it is appropriate for a vaccine distribution program to prioritize its own citizens/residents over foreign citizens located in other countries. While pragmatic interests may support distributing the vaccine globally (e.g., to prevent transnational spread) or in advance of some other ethical obligation (e.g., charity or benevolence), inequity among nation‐states is not itself unjust.

Critics argue that the vaccine nationalist approach ignores the equal worth of humans and leads to morally unconscionable results.^3^ While a nation‐state might elect to distribute vaccines internationally out of self‐interest (e.g., to avoid variants or contain transmission), this same self‐interest and preference for its citizens permits hoarding of vaccines, even if it results in widespread death beyond its borders.

## 4 Hybrid Approaches

Some critics of both “pure” vaccine nationalism and vaccine cosmopolitanism advocate for a hybrid‐type approach. One account of the hybrid model was presented by Ferguson and Caplan [9] who argued that conflicting duties are formed by the “principle of equality” and the “obligation to prioritize one's own.” Ferguson [9, p. 3] explains, in line with the cosmopolitan view, that the principle of equality is a general obligation based on the equal worth of humans who are equally deserving of “benevolence.” However, because individuals live in the context of non‐global communities (e.g., nation‐states), special obligations arise among community members (e.g., gratitude and reparation), created by “associational ties [9].” Ferguson and Caplan [9, p. 1] conclude that a “limited nationalism” that is responsive to such associational moral commitments can complement general egalitarian obligations, rather than present an obstacle.

In the context of the COVID‐19 crisis, Ferguson and Caplan [9, p. 3] concede, in line with a cosmopolitan view, that all persons deserve “health and protection from the coronavirus.” However, within a nation‐state, there are also “legitimate moral duties to procure and allocate vaccines in a self‐interested manner [9].” Ferguson and Caplan [9, p. 2] argue that “cosmopolitan obligations do not *automatically* outweigh obligations one bears by virtue of one's membership to other, smaller scope communities.” (Emphasis mine) Ferguson and Caplan argue that just distribution of vaccines can, and should, accommodate both of these conflicting commitments. They endorse a “*limited* national partiality” in vaccine distribution [9] but explicitly decline to prescribe a specific vaccine allocation regimen using the hybrid model. (emphasis mine) [9, p. 4]

Emmanuel et. al. [3, p. 543] argue in favor of a hybrid model, the Fair Priority for Residents (FPR), that provides more specificity of how vaccines should be distributed domestically and internationally. Emmanuel et. al. reject what they describe as “radical cosmopolitanism” and “radical nationalism” for reasons similar to those offered by Caplan and Ferguson. According to Emmanuel et. al., radical cosmopolitanism ignores a government's “special obligations” to care for its citizens’ fundamental interests, including health [3, p. 4]. Radical nationalism is rejected because it permits or obliges countries to seek “full normalcy” before offering vaccines internationally, which “violates equal respect for individuals [3].” The FPR model is a compromise which requires equitable international distribution of resources, after domestic distribution achieves noncrisis level of disease, which it benchmarks at historic flu‐level mortality [3, p. 5]. The FPR model further provides that international distribution will be guided by the mortality levels in various states [3, p. 9]. Nation states with the greatest vulnerability to disease will receive additional resources to curb mortality.

The hybrid models of Ferguson and Caplan and Emanuel et al., endorse some form of “limited nationalism.” Both accounts endorse the view that moral obligations based in domestic associative relationships will permit some level of priority to nationals in pandemic vaccine distribution. Limited nationalism models acknowledge that prioritization will generally favor high income countries (HICs), which can more easily secure vaccines, and disfavor low to middle income countries (LMICs), who cannot. Limited nationalism further accepts that during the domestic prioritization period, LMICs countries will have relatively high mortality rates compared to HICs.

## 5 Global Interdependence and Disease

While cosmopolitans, nationalists, and hybrid proponents generally agree that social cooperation and/or interdependence (referred collectively hereinafter as “interdependence”) are important components for establishing distributive justice obligations, they diverge on what interdependence level is necessary. Since the early global justice debates between cosmopolitans and nationalists, evidence linking interdependence on the welfare of specific states has dramatically increased. Such interdependence significantly impacts the underlying conditions which foster the emergence, transmission, and ability to remediate mortality both within specific nation‐states and globally in the case of a pandemic.

As to the general welfare of states, past and present geopolitics and geoeconomics, particularly between LMICs and HICs, create and perpetrate socioeconomic advantages and disadvantages [4]. Historic harms, like colonization, the global slave trade, and predatory trade practices have demonstrable ripple effects that continually provide economic benefit to HICs and disadvantage LMICs [4]. Many of these current socioeconomic dis/advantages themselves are social determinants of the pre‐pandemic health within nation‐states, which then shape how nation‐states will fare during pandemics.

### 5.1 Disease Emergence

Ecological interdependence, which absent from many just vaccine distribution discussions, has played a unique and significant role in the emergence and transmission of multiple recent endemics and pandemics, including COVID‐19 [11]. The prevailing origin theory is that the pathogen that causes COVID‐19 is zoonotic—a virus that transmits from a host species to humans [11]. This paper will presume that this origin account is accurate. The presumption is prospectively instructive as most emerging diseases are zoonoses and the next pandemic will likely be caused by a zoonotic virus [12].

Zoonotic viruses frequently emerge during ecological changes. For humans, these changes include “changing travel or immigration patterns, intensified agricultural practices, changing land use patterns and encroachment into wildlife habitat, climate change in human occupied areas and range expansion of virus vectors or reservoirs [12].” Such changes are often the result of broader, socioeconomic global forces. HICs have a disproportionate influence on these global forces and generally reap the lion share of benefits, while LMICs bear the greater burdens [13]. For example, HICs are a disproportionate contributor to greenhouse gases that result in global warming [13]. However, LMICs, particularly in the global south, are disproportionately burdened by the effects of global warming [13]. Rising temperatures linked to global warming have played a central role in the emergence of recent zoonotic viruses.^4^

Similarly, agricultural practices and changing land use patterns, which cause encroachment into wildlife habitats, are often attributable, to some degree, to both historic and current economic/political colonialism and/or predatory trade practices. For example, Western nations aggressive trade practices against LMICs within the global south resulted in lopsided economies, frequently called banana republics, which stunted broader economic development in LMICs. Governments of LMICs will often permit ecologically unsustainable development to “catch up” to HICs. Such encroachment will bring human populations in closer and more consistent contact with latent virus reservoirs within previously remote/isolated species.

### 5.2 Disease Transmission

Once a zoonotic virus emerges in a human host, the spread and casualty rate of the virus depends on interaction with people whose underlying health and social conditions create pathways for disease spread [11, p. 30]. In LMICs, pathways for transmission are created by widespread poverty, lack of access to basic health care resources, poor nutrition, poor sanitation, crowded living and working conditions and co‐occurring diseases like HIV/AIDS and malaria [11, p. 30]. In HICs, transmission pathways follow economic inequalities and associated poverty, lack of health insurance and/or access to affordable care, obesity, congregate social gatherings, aging populations, and comorbid diseases like diabetes and heart disease [11, p. 30]. This divide between LMIC and HIC domestic transmission pathways highlights the link between interdependence, and the downstream effects of inequality and health.

Looking beyond domestic transmission, modern social interconnectedness characterized by more international travel, urbanization, and international commerce leads to more rapid transmission of disease globally. While international mobility and commerce can impart socioeconomic benefits for both HICs and LMICs, HICs have historically been able to receive greater advantages from global cooperation/interconnectedness [14]. For example, the United Nations Conference on Trade and Development states that “market distortions” by HICs cost the developing world 700 billion dollars annually [14].

### 5.3 Disease Remediation and Mortality

Interdependence negatively impacts both the levels of mortality and ability to remediate disease of LMICs relative to HICs. Interdependence can negatively impact both the underlying health of LMIC populations making them more vulnerable to the ravages of disease. It can also hinder broad socioeconomic development, including the healthcare resources, both personnel and infrastructure, needed to remediate disease. During the COVID‐19 pandemic, LMICs lacked the technological resources to treat infected populations. In early 2020, LMICs had a paucity of ICU beds and ventilators relative both to HICs and the size of their populations [14]. Access to clean water and soap was a struggle for many LMICs [14]. While nearly every country struggled with medical staffing shortages, LMICs were hit particularly hard, due to decades of “brain drain” wherein “healthcare workers in HICs received training in lower income nations and providing substantial benefits to their adopted nations at the expense of their home nations[.] [14]” Finally, LMICs were ill‐equipped to develop, procure, and disseminate vaccines. For example, “in addition to domestically produced vaccines, the United States purchased 1.2 billion vaccines from external sources, which could cover even more than twice [its] population [15].” In contrast, some LMICs, like Moldova and Albania, which lack capacity to domestically develop vaccines “have only purchased enough vaccines to cover 5% of their population [15].”

The interdependence between nations on so many fronts has led some commentators to describe the COVID‐19 crisis as a syndemic, rather than a pandemic [11, p. 28]. A syndemic occurs when biosocial forces converge and interact with one another to produce and exacerbate clinical disease and prognosis [11, p. 28]. This framework provides insight into the synergistic features of diseases where there is “social, economic, environmental and political milieu in which a population is immersed [11, p. 28].” It also reveals how “individual health is increasingly linked to population health, both within and between countries [11, p. 28].”

Applying to the Singer illustration these observations on the effects of global interdependence and its dynamic relationship to disease creates a different picture. Incorporating the effects of global interdependence, Peter is no mere bystander. Peter may have directly or indirectly caused or increased the peril faced by the drowning child. Certainly, if Peter was directly or indirectly, partially or wholly, responsible for either the child's placement in the pond, or the perilousness (e.g., depth/breadth) of the pond, Peter's moral obligation to provide aid is significantly altered. The moral obligation to rescue takes on an additional compensatory justice component that changes Peter's calculation of what may be required of him to rescue the child.

## 6 Interdependence and Compensatory Justice, a New Hybrid Model Proposal

The limited nationalism hybrid models presented in the Ferguson and Caplan article and the Emanual et al. article have multiple advantages over both the “pure” cosmopolitan and nationalist accounts. Their models improve on the cosmopolitan model by providing a more complete account of the moral landscape of both individuals and nation‐states—simultaneously embracing the moral obligation of membership in the global community as well as the important moral obligations associated with domestic community memberships. In embracing international justice obligations, such hybrid models avoid the result of resource hoarding and indifference to human suffering from pure nationalist accounts.

However, neither limited nationalism model examined is consistent with an understanding of dis/advantages caused by interdependence and its relationship to disease, particularly when the disease results in a syndemic. These limited nationalist hybrid models acknowledged the disparate health prospects of LMICs and HICs, however they neglected to consider the full impact of historic and current injustices perpetrated by interactions among nations. While such limited nationalism models may be instructive in assessing justice obligations either between nation‐states on equal geopolitical/economic footing or between LMICs and HICs whose history avoided meaningful interdependence, they fail to address the moral contours of global interdependence as it impacts many nation‐states.

Because interdependence is extensive, playing a significant part in shaping the health outcomes of nations and individuals, any vaccine distribution model that ignores interdependence will be either ahistorical or morally incomplete. A conception of international distributive justice should comport with a robust and historically accurate understanding of interdependence. Where interdependence shows that HICs have significantly and detrimentally impacted the prospects of LMICs, additional cosmopolitan‐leaning moral considerations will factor into a just vaccine distribution model.

Compensatory justice, to the extent that it rectifies or offsets historic harms and current vulnerabilities of LMICs, will be an important part of the moral calculation of any distributive justice model. The application of compensatory justice to the international context is not new. Thomas Pogge, for example, argues that the sharp wealth disparity between countries is demonstrably attributable to a “common and very violent history,” citing specifically the ravages of colonialization of LMICs by HICs. Based on historic wrongs, Pogge concludes that HICs have a moral obligation based on justice, not generosity, to provide aid to LMIC. Whereas Pogge focused principally on rectifying poverty through compensatory justice, its role in the distribution of vaccines is a reasonable extension of Pogge's position given the broad nature of harms and their link to the health of LMIC populations. Absent compensatory justice, HICs will simply be able to exploit their advantage moving forward with impunity.

The specific epidemiology of the pandemic may make a limited nationalism theory even more morally untenable. For example, the necessity of ventilators as a medical intervention to address the COVID‐19 virus, meant that HICs prioritizing domestic distribution resulted in significantly higher rates of mortality in many LMICs that had an insufficient supply of this technology. This inequity is aggravated if an HIC, perhaps because its pandemic mortality rate has not dipped below emergency levels, elects to distribute vaccines to their less vulnerable population before allocating internationally.

Critically, a hybrid vaccine distribution model that is appropriately sensitive to interdependence may not necessarily favor priority to nationals, i.e., limited nationalism, as Ferguson and Caplan and the Emanual et al. assert. While limited nationalism proponents may be correct that cosmopolitan obligations should not *automatically* outweigh community obligations, any interdependence associated with a pandemic, particularly one that has disparate health impacts on LMICs, will likely create weighty compensatory justice obligations. Accordingly, a vaccine distribution model should follow a diagnostic of relevant effects of interdependence and the pandemic epidemiology. A properly calibrated hybrid model, which takes into account interdependence, historical harms, and the disproportionate impact of the pandemic on LMICs, may even preference initial vaccine distribution into LMICs *before* HICs, which might be described as limited cosmopolitanism.

The Singer model continues to be illustrative. Peter sees two children drowning. Peter has some associational link to one child but no associational link to the other. However, Peter was, in some measure, complicit in the other child's predicament. While both children are in some peril of drowning, the child that Peter knows is in much shallower water and near a flotation device. The child that Peter knows will most likely experience some significant distress, but the unknown child faces a significant chance of death.

## 7 Conclusion

The COVID‐19 pandemic exposed how interdependence helped shape the pandemic and intertwined the health of countries. A review of competing vaccine distribution models strongly favors a hybrid account that is responsive to interdependence and factors in compensatory justice considerations. At most, under a hybrid model, domestic community obligations *might* permit HICs to prioritize an initial domestic distribution of vaccines to healthcare workers and the most vulnerable before a similar allocation to LMICs. However, I am not convinced that even this “modest” level of nation‐state preference would afford proper weight to the competing moral obligations of most HICs in the context of the last pandemic or future ones. Such obligations will likely overwhelm the competing obligations based on nationalism.

## Conflicts of Interest

The author declares no conflicts of interest.

## Endnotes

1 Emanuel et al. [7, p. 1309]. Notably, the authors indicate some reluctance endorsing initial domestic distribution as ethical. However, they state that after the rate of transmission falls below 1, domestic distribution can no longer be justified.

2 Caney [1, p. 980]. *See* Emanuel et al. [7, p. 1309] discussing nationalism emphasis on “associative” ties developed within nations. *See also* Ferguson and Caplan [9, p. 4].

3 Ferguson and Caplan [9] describing “bad nationalism.”

4 Dennehy [12, p. 137] citing the Blue Tongue Virus and the Hantavirus.
